# Supplementary material for: Influence of Dietary n-3 Long Chain Polyunsaturated Fatty Acid Intake on Oxylipins in Erythrocytes of Women with Rheumatoid Arthritis
Source: Molecules. 2023 Jan 11;28(2):717. doi: 10.3390/molecules28020717 (PMC9863541; doi:10.3390/molecules28020717)
Supplement: Supplementary file 1 [file molecules-28-00717-s001.zip › molecules-2045398-supplementary.pdf]

Table S1. Erythrocyte free fatty acids correlations to erythrocyte oxylipins.

	Free Arachidonic Acid		Free Adrenic Acid		Free EPA		Free DHA	
	r	*p*	r	*p*	r	*p*	r	*p*
Tetranor−PGFM	−0.083	0.706	−0.04	0.858	−0.124	0.574	−0.116	0.599
12−HHTrE	0.274	0.206	0.303	0.16	0.23	0.29	0.238	0.273
11−HETE	0.097	0.66	0.207	0.344	0.145	0.508	0.123	0.578
9−HETE	−0.212	0.33	−0.196	0.371	−0.067	0.76	−0.127	0.564
5−HETE	0.293	0.174	0.333	0.121	0.373	0.08	0.38	0.073
15−HETE	0.184	0.401	0.174	0.427	0.228	0.295	0.209	0.337
5,15−diHETE	0.1	0.65	0.13	0.556	0.119	0.59	0.14	0.523
8−HETE	0.470*	0.024	0.440*	0.036	0.497*	0.016	0.441*	0.035
12−HETE	0.132	0.547	0.191	0.383	0.23	0.291	0.251	0.248
19−HETE	−0.199	0.363	−0.24	0.269	−0.26	0.231	−0.29	0.179
11(12)−EET	0.646**	<0.001	0.610**	0.002	0.567**	0.005	0.517*	0.011
11,12−diHETErE	0.336	0.117	0.418*	0.047	0.25	0.251	0.344	0.108
14(15)−EET	0.442*	0.035	0.430*	0.041	0.348	0.103	0.347	0.105
14,15−diHETrE	0.438*	0.037	0.517*	0.012	0.264	0.224	0.339	0.114
5,6−diHETrE	0.387	0.068	0.481*	0.02	0.335	0.096	0.427*	0.042
9−HEPE	0.265	0.222	0.139	0.528	0.454*	0.029	0.334	0.119
11−HEPE	0.274	0.206	0.297	0.169	0.381	0.073	0.3	0.165
18−HEPE	0.135	0.54	0.143	0.515	0.238	0.275	0.169	0.441
15−HEPE	0.084	0.703	0.111	0.615	0.206	0.346	0.081	0.713
12−HEPE	0.081	0.714	0.031	0.889	0.12	0.585	0.046	0.833
4−HDoHE	0.184	0.4	0.133	0.546	0.335	0.118	0.272	0.209
8−HDoHE	0.2	0.361	0.265	0.222	0.22	0.313	0.239	0.273
16−HDoHE	0.295	0.171	0.356	0.096	0.404	0.056	0.417*	0.048
20−HDoHE	0.203	0.352	0.229	0.292	0.31	0.12	0.346	0.106
13−HDoHE	−0.013	0.953	−0.034	0.878	−0.067	0.762	0.005	0.982
10−HDoHE	0.201	0.953	−0.034	0.878	−0.067	0.762	0.005	0.982
14−HDoHE	0.558**	0.006	0.586**	0.003	0.547**	0.007	0.528**	0.01
16(17)−EpDPE	0.419*	0.047	0.318	0.139	0.554**	0.006	0.501*	0.015
15−HETrE	0.031	0.89	−0.01	0.964	0.108	0.625	0.063	0.774
9−HOTrE	0.435*	0.038	0.453*	0.03	0.462*	0.027	0.444*	0.034
13−HODE	0.093	0.673	0.119	0.59	0.208	0.34	0.186	0.396
9−HODE	0.105	0.634	0.119	0.59	0.208	0.34	0.186	0.396
9−oxoODE	−0.082	0.71	0.075	0.733	−0.011	0.961	−0.07	0.75

9(10)−EpOME	−0.046	0.833	0.024	0.914	0.048	0.83	−0.023	0.918
9,10−diHOME	−0.04	0.858	−0.08	0.717	0.082	0.71	−0.037	0.868
12(13)−EpOME	−0.053	0.809	0.068	0.757	−0.079	0.72	−0.064	0.771
12,13−diHOME	−0.071	0.747	−0.166	0.449	0.033	0.883	−0.124	0.574
Free Arachidonic Acid			0.944**	<0.001	0.940**	<0.001	0.944**	<0.001
Free Adrenic Acid	0.944**	<0.001			0.859**	<0.001	0.908**	<0.001
Free EPA	0.940**	<0.001	0.859**	<0.001			0.944**	<0.001
Free DHA	0.944**	<0.001	0.908**	<0.001	0.944**	<0.001		

* *p*<0.05, ***p*<0.01, Spe arman correlation cofficients and *p*−values for free fatty acids and oxylipins.

Oxylpins: prostaglandin F Metabolite (PGFM), hydroxyheptadecatrienoic acid (HHTre), hy−droxyoctadeca trienoic acid (HOTrE), epoxyoctadecenoic acid (EpOME), dhydroxy−octadecenoic acid (diHOME), hydroxyoctadecadienoic acid (HODE), hydroxyeicosapentaenoic acid (HEPE), hydroxydocosahexaenoic acid (HDoHE), hydroxyeicosatrienoic acid (HETrE), dihydroxyeicosa−trienoic acid (di−HEIrE), oxo−octadecadienoic acid (oxoODE), hydroxyeicosatetraenoic (HETE), dilhydroxyeicosatetraenoic acid (diHETE), epoxy−eicos atrienoic acid (EET), epoxy−docosapentaenoic acid (EpDPE).

Table S2. Erythrocyte free fatty acids(%) correlations to erythrocyte oxylipins.

	Linoleic acid (18:2n−6)		α −linolenic acid (18:3 n−3)		Linoleic acid (20:3 n−6)		Arachidonic acid (20:4 n−6)		Eicosapentaenoic acid (20:5 n−3)		Docosahexaenoic acid (22:6 n−3)	
	r	*p*	r	*p*	r	*p*	r	*p*	r	*p*	r	*p*
Tetranor−PGFM	0.107	0.628	0.193	0.378	−0.208	0.342	0.255	0.24	0.034	0.879	−0.383	0.071
12−HHTrE	−0.364	0.088	0.095	0.666	−0.508*	0.013	0.196	0.37	0.047	0.83	0.014	0.95
11−HETE	−0.114	0.606	0.357	0.095	−0.152	0.488	−0.231	0.288	−0.081	0.713	−0.089	0.687
9−HETE	−0.155	0.48	0.243	0.264	−0.023	0.918	−0.251	0.248	−0.043	0.847	−0.209	0.339
5−HETE	−0.169	0.441	0.289	0.2	−0.102	0.644	−0.329	0.125	−0.024	0.914	0.18	0.412
15−HETE	−0.347	0.105	0.264	0.224	−0.299	0.165	−0.039	0.861	0.031	0.89	−0.01	0.964
5,15−diHETE	0.049	0.832	0.313	0.146	−0.412	0.05	−0.286	0.186	0.28	0.196	0.221	0.312
8−HETE	−0.469*	0.024	0.183	0.403	−0.22	0.314	0.072	0.743	0.272	0.209	0.073	0.74
12−HETE	−0.384	0.07	0.181	0.409	−0.077	0.727	−0.03	0.893	0.187	0.394	−0.076	0.73
19−HETE	0.213	0.329	−0.431*	0.04	0.219	0.315	0.107	0.627	−0.24	0.269	0.069	0.753
11(12)−EET	−0.4	0.058	0.06	0.785	0.001	0.996	0.451*	0.031	0.216	0.321	−0.075	0.73.5
11,12−diHETErE	−0.538* *	0.008	−0.099	0.652	−0.234	0.281	0.462*	0.026	*0.02	0.928	0.001	0.996
14(15)−EET	−0.444*	0.034	−0.340	0.158	−0.09	0.682	0.422*	0.045	0.079	0.72	−0.074	0.737
14,15−diHETrE	−0.474*	0.022	−0.182	0.406	−0.048	0.826	0.478*	0.021	−0.148	0.5	−0.125	0.568
5,6−diHETrE	−0.331	0.122	−0.029	0.897	0.246	0.257	−0.027	0.904	−0.148	0.5	−0.125	0.612
9−HEPE	−0.276	0.202	0.445*	0.033	−0.349	0.102	−0.25	0.251	0.529**	0.009	0.306	0.156
11−HEPE	−0.31	0.15	0.191	0.383	−0.408	0.053	−0.312	0.149	0.489*	0.018	0.461*	0.027
18−HEPE	−0.189	0.389	0.26	0.231	−0.33	0.124	−0.326	0.128	0.409	0.053	0.142	0.519
15−HEPE	−0.186	0.396	0.128	0.562	−0.145	0.508	−0.192	0.38	0.094	0.67	0.129	0.559
12−HEPE	−0.274	0.207	0.268	0.215	−0.245	0.259	−0.189	0.388	−0.002	0.995	−0.029	0.869
4−HDoHE	−0.367	0.085	0.393	0.063	−0.525*	0.01	−0.105	0.635	0.475*	0.022	0.197	0.367
8−HDoHE	−0.128	0.56	−0.085	0.699	−0.272	0.209	−0.051	0.819	−0.252	0.247	0.088	0.689
16−HDoHE	−0.420*	0.046	−0.089	0.686	−0.166	0.448	0.106	0.63	0.187	0392	0.174	0.727
20−HDoHE	−0.420*	0.046	−0.089	0.686	−0.166	0.448	0.106	0.63	0.187	0.392	0.174	0.426
13−HDoHE	0.042	0.848	0.203	0.352	−0.348	0.103	0.007	0.975	0.084	0.704	−0.234	0.283
10−HDoHE	−0.503*	0.014	−0.052	0.815	−0.151	0.492	0.481*	0.02	0.231	0.288	−0.131	0.551
14−HDoHE	−0.439*	0.036	0.085	0.701	−0.269	0.215	0.05	0.822	0.256	0.239	0.222	0.309
16(17)−EpDPE	−0.424*	0.044	0.173	0.43	−0.404	0.056	−0.178	0.417	0.420*	0.046	0.368	0.084
15−HETrE	−0.181	0.409	0.025	0.911	0.016	0.943	−0.055	0.802	−0.166	0.449	−0.073	0.74
9−HOTrE	−0.22	0.312	0.332	0.122	−0.304	0.158	0.004	0.986	0.381	0.073	0.047	0.832
13−HODE	−0.065	0.768	0.486*	0.019	−0.164	0.455	−0.347	0.105	0.021	0.925	−0.048	0.826
9−HODE	−0.11	0.618	0.512*	0.013	−0.089	0.687	−0.193	0.378	0.022	0.922	−0.267	0.218
9−oxoODE	0.114	0.606	0.461*	0.027	0.263	0.226	−0.123	0.578	−0.193	0.378	−0.354	0.098

9(10)−EpOME	0.077	0.727	0.535**	0.009	−0.186	0.396	−0.4	0.058	0.043	0.844	−0.127	0.562
9,10−diHOME	0.378	0.075	0.391	0.065	0.15	0.494	−0.274	0.206	−0.462	0.46	−0.123	0.578
12(13)−EpOME	0.122	0.581	0.373	0.08	0.002	0.993	−0.33	0.124	−0.232	0.286	−0.148	0.5
12,13−diHOME	0.096	0.664	0.570**	0.005	−0.053	0.809	−0.294	0.173	−0.123	0.578	−0.157	0.474
Linoleic acid (18:2n−6)			0.204	0.352	0.202	0.356	−0.430*	0.041	−0.365	0.087	−0.157	0.474
α −linolenic acid (18:3 n−3)	0.204	0.352			0.008	0.971	−0.432*	0.04	0.212	0.33	−0.148	0.5
Linoleic acid (20:3 n−6)	0.202	0.356	0.008	0.971			0.142	0.517	−0.358	0.094	−0.286	0.187
Arachidonic acid (20:4 n−6)	−0.430*	0.041	−0.432*	0.04	0.142	0.517			−0.233	0.284	−0.447*	0.033
Eicosapentaenoic acid (20:5 n−3)	−0.365	0.087	0.212	0.33	−0.358	0.094	−0.233	0.284			0.535**	0.009
Docosahexaenoic acid (22:6 n−3)	−0.157	0.474	−0.148	0.5	−0.286	0.187	−0.447*	0.033	0.535**	0.009		

*$p < 0.05$, ** $p < 0.01$, Speam an cxrEadian coficients and p−values for freefaty acids and ayipins. Oay lipins:prostaglandin F Metabolite(PGFM), hydrowyheptadcadrinaic acid (HHTe), hy−droayoctadecatrianoic aid (HOTnrE), eponyocdadecendic acid(EpOME), cdihy droy−octadecanoic acid (dHOME) hy droryoctadecadianoic acid (HODE) iny dronyeicosapeantanoic acid (HEPE), hydroy docosahexanoic ad (HDoHE,hydronyeicosatriendicacid (HETrE),dihydronyeicsa−tiendic acid(d−HET1E), oxo−odtadecadienc acid(axcODE),hydrouy eiccsatderanoi (HETE)dtnydrow icsaetraenacacid(diHETE), epooy −eicosatriencic acil (EET), epaxy−docosapentaenoic acid EpDPE). *$p < 0.05$, ** $p < 0.01$
